# The etiology and differential diagnosis of “autoimmune hepatitis-like liver disease” in children: a single-center retrospective study

**DOI:** 10.3389/fped.2024.1377333

**Published:** 2024-05-16

**Authors:** Di Ma, Xinglou Liu, Guo Ai, Wen Pan, Lingling Liu, Yuan Huang, Yi Liao, Yuanyuan Lu, Zhan Zhang, Hua Zhou, Zhihua Huang, Xingjie Hao, Sainan Shu, Feng Fang

**Affiliations:** ^1^ Department of Pediatrics, Tongji Hospital, Tongji Medical College, Huazhong University of Science and Technology, Wuhan, China; ^2^ Department of Epidemiology and Biostatistics, Ministry of Education Key Laboratory of Environment and Health, Tongji Medical College, Huazhong University of Science and Technology, Wuhan, China

**Keywords:** children, autoimmune hepatitis, serum globulin, immunoglobulin G, autoantibodies, histology

## Abstract

**Background:**

Children with autoimmune hepatitis (AIH) often present with symptoms similar to those of other liver diseases. This study consists of a comparison between the clinical and histological characteristics of AIH and those of other four AIH-like liver diseases [i.e*.*, drug-induced liver injury (DILI), gene deficiency, infectious liver disease and other etiology of liver disease], as well as an evaluation of the AIH scoring system's diagnostic performance.

**Methods:**

All children with AIH-like liver disease at our center from January 2013 to December 2022 were included. The clinical and histological characteristics of the AIH group were retrospectively analyzed and compared with those of the other four groups.

**Results:**

A total of 208 children were included and divided into AIH group (18 patients), DILI group (38 patients), gene deficiency group (44 patients), infectious liver disease group (74 patients), and other etiology group (34 patients). The antinuclear antibodies (ANA) ≥ 1:320 rate was significantly higher in the AIH compared to the other four groups after multiple testing correction (*p *< 0.0125), while patients with positive antibodies to liver-kidney microsomal-1 (anti-LKM1, *n* = 3) and smooth muscle antibodies (SMA, *n* = 2) were only observed in the AIH group. The positive rates of antibodies to liver cytosol type1 (anti-LC1) and Ro52 were higher than those in the other four groups. The serum immunoglobulin G (IgG) and globulin levels, as well as the proportions of portal lymphoplasmacytic infiltration, lobular hepatitis with more than moderate interface hepatitis, and lobular hepatitis with lymphoplasmacytic infiltration, were significantly higher in the AIH group than in the other four groups after multiple testing correction (*p *< 0.0125). The cirrhosis rate in the AIH group was higher than that in the DILI and infectious liver disease groups (*p *< 0.0125). Both the simplified (AUC > 0.73) and the revised systems (AUC > 0.93) for AIH have good diagnostic performance, with the latter being superior (*p *< 0.05).

**Conclusion:**

Positive autoantibodies (ANA ≥ 1:320 or anti-LKM1 positive, or accompanied by SMA, anti-LC1 or Ro-52 positive) and elevated serum IgG or globulin levels contribute to early recognition of AIH. The presence of lobular hepatitis with more than moderate interface hepatitis and lymphoplasmacytic infiltration contribute to the diagnosis of AIH.

## Introduction

AIH is an inflammatory liver disorder with no clear cause which may be associated with various factors such as genetics, immunity, and the environment (including infection or exposure to toxins) (1). The diagnosis of AIH is based on histological abnormalities (e.g., interface hepatitis, lymphoplasmacytic infiltration and emperipolesis), autoantibodies, and other characteristic laboratory findings (e.g., elevated serum aspartate aminotransferase (AST) and alanine aminotransaminase (ALT), and increased IgG concentration) (2).

The diagnosis of AIH is rather challenging due to the absence of a signature diagnostic marker. AIH-related autoantibodies also appear in acute liver injury caused by other etiologies. Additionally, some liver diseases, including chronic viral hepatitis, may also lead to elevated levels of serum globulin or IgG (3)_._ Dutch scholars have found that 40% of patients with DILI have elevated serum IgG levels, and the positive rates for ANA and antimitochondrial antibodies (AMA) are between 60% and 70%. However, the titers of these autoantibodies tend to decrease within several months (4). In a pediatric metabolic dysfunction-associated fatty liver disease (MAFLD) cohort, one-third of patients were positive for autoantibodies (5). Primary biliary cholangitis (PBC) are often accompanied by positive autoantibodies such as AMA and ANA (6). Krithiga et al. reported a case of Epstein-Barr virus hepatitis with the complication of Hodgkin's lymphoma in which the patient had likely been misdiagnosed and treated for AIH in the index presentation (7).

In this article, we define liver disease with biochemical characteristics of AIH and/or positive autoantibodies as “AIH-like liver disease”, collecting patients' clinical, biochemical, immunologic and histologic data. Moreover, we will compare the clinical and histological features of AIH with those of other AIH-like liver diseases, and evaluate the diagnostic performance of the AIH scoring system.

## Materials and methods

### Study design and patients

This retrospective study was performed in the Department of Pediatrics at Tongji Hospital between January 2013 and December 2022. All participants met the following conditions: (1) signs of liver damage, including unexplained elevation of aminotransferases, hepatomegaly, or cirrhosis; (2) meeting one or more of the following criteria: unexplained elevation in serum globulin (>35 g/L); unexplained elevation in IgG [exceeding the upper limit of the normal range for the same age group. (0–28 days: 6.6–17.5 g/L; 29 days-6 months: 2.0–6.9 g/L; 6 months-3 years: 3.3–12.3 g/L; 3–12 years: 5.4–15.3 g/L; 12–17 years: 7.0–15.6 g/L)]; positive for at least one autoantibody; (3) diagnosis at <18 years of age.

The exclusion criteria were: (1) patients with extra-hepatic autoimmune diseases such as systemic lupus erythematosus and rheumatoid arthritis at the time of initial diagnosis; (2) patients with severe systemic or extra-hepatic diseases, such as severe cardiopulmonary disease and severe sepsis; (3) patients who had received intravenous immunoglobulin therapy within the 12 weeks before initial diagnosis; (4) patients with biliary obstruction diseases such as biliary calculi and biliary atresia; (5) patients with severe clinical data deficiencies; (6) diagnosis at ≥18 years of age.

The study protocol was approved by the Medical Ethics Committee at Tongji Hospital (no.: TJ-IRB20230302), and it adhered to the tenets of the Declaration of Helsinki. Written informed consent was obtained from the parents of all included patients. Clinical and laboratory data were obtained from the patients' medical records.

Diagnostic criteria for the etiology of AIH-like liver disease were: (1) AIH (2): patients conformed with the 1999 revised International Autoimmune Hepatitis Group (IAIHG) score (8) ≥16 and/or 2008 IAIHG simplified AIH score (9) ≥7, and/or histological features consistent with AIH. These included: (i) more than moderate interface hepatitis (mild: local or few portal areas destroyed; moderate: <50% of portal areas or fibrous septa destroyed; severe: >50% of portal areas or fibrous septa destroyed); (ii) lymphoplasmacytic infiltration with plasma cells accounting for ≥20% of inflammatory cells, and/or focal plasma cell clustering in the portal or lobule (defined as >5 plasma cells in one focus); (iii) hepatocellular rosettes; (iv) emperipolesis; (v) necroinflammation in the central lobules. Moreover, treatment with corticosteroid or immunomodulators and long-term follow-up (>1 year) were necessary for diagnosis. (2) DILI (10): history of using hepatotoxic drugs regardless of whether the dose reached toxic levels; recovery from liver disease after symptomatic treatment, or accepted corticosteroids therapy less than six months and no recurrence during long-term follow-up (>1 year). (3) Infectious liver disease (11): existing etiological evidence of hepatitis virus infection or other viral active infection, or existing clinical and etiological evidence of systemic infection. (4) Gene deficiency diseases including either hepatolenticular degeneration [according to the Leipzig Diagnostic Criteria (12) and in concordance with the American Association for the Study of Liver Diseases (AASLD) guidelines (13)]. or other genetic liver diseases which can only be diagnosed by genetic testing, including progressive familial intrahepatic cholestasis (PFIC). (5) Other etiology of AIH-like liver diseases including either PBC (14) (according to the AASLD practice guidelines) or MAFLD (15): pathological or imaging evidence of intrahepatic fat accumulation and one of the following three items: excessive obesity, prediabetes or type 2 diabetes, and metabolic disorders.

### Data collection

The collected information included general data (e.g., age, sex), clinical manifestations (e.g., jaundice and fatigue), laboratory examinations, imaging data (to confirm cirrhosis and rule out any obstructive liver injury etiology), pathological data, and the treatment outcomes of the children meeting the above selection criteria.

### Laboratory evaluations

The laboratory evaluations included tests for pathogens (e.g., hepatitis A–E viruses, Epstein‒Barr virus, cytomegalovirus), liver function markers (i.e., ALT, AST, total bilirubin (TB), direct bilirubin (DB), alkaline phosphatase (ALP), gamma-glutamyl transpeptidase (GGT), lactate dehydrogenase, serum albumin, globulin, cholesterol), blood routine, coagulation function (prothrombin time (PT), international normalized rate (INR)) and IgG.

### Autoantibodies

The antibody spectrum includes ANA, anti-LKM1, AMA-M2, AMA-M2-3E, anti-LC1, antibodies to soluble liver antigen (anti-SLA), nuclear membrane glycoprotein 210 antibodies (GP210), SP100 nuclear antigen antibodies (SP100), Ro-52, and SMA. Our hospital has adopted an autoantibody kit that is compatible with the indirect immunofluorescence analyzer and immunoblotting analyzer (all from EUROIMMUN Medizinische Labordiagnostika AG, Lübeck, Germany). Among them, ANA and SMA are detected by indirect immunofluorescence, with a cut-off value of 1:100 [due to the different dilution systems, the detection of ANA and SMA at 1:100 in this system is equivalent to the lowest titer (1:40) in the AIH score systems]; the remaining antibodies are detected by immunoblotting (EUROLineScan color intensity 11–25 is weakly positive, 26–50 is positive).

### Clinical outcome

The outcomes included four categories (1) Recovery: normalization of blood biochemical indicators, with no recurrence observed within six months following treatment cessation; (2) Chronicity: Liver function indicators remained abnormal after 6 months of the course of the disease, or there was evidence of chronic liver disease on imaging or histology; (3) Cirrhosis: Liver imaging and/or histopathological changes in liver tissue met the characteristics of cirrhosis; (4) Death.

### Statistical analysis

All statistical analyses were performed with SPSS v. 26.0. The Kolmogorov–Smirnov test was used to verify whether the variables followed a normal distribution. Continuous variables with normal distributions were expressed as mean ± standard deviation and analyzed by one-way analysis of variance (ANOVA) test or student's *t*-test for pair-wise groups. Continuous variables with skewed distributions were expressed as medians [interquartile range (IQR)] and analyzed by a Kruskal–Wallis test and a Wilcoxon rank-sum test for pair-wise groups. Categorical variables were expressed as absolute number (percentage), and evaluated using either a *χ*^2^ or Fisher's exact test. The revised and simplified systems' diagnostic accuracy was evaluated with the receiver operating characteristic (ROC) curve. The difference in the ROC curves for the revised and simplified systems were judged with a DeLong test. For each test, *p *< 0.05 was considered statistically significant. Bonferroni correction was used when multiple tests were conducted for pair-wise comparison.

## Results

### Basic characteristics for patients with AIH-like liver disease

A total of 208 children with AIH-like liver disease were included, among the 3,852 children with liver injury in this study. The median age was 52.5 (17.0, 98.0) months, with 104 boys and 104 girls, indicating a balanced male-to-female rate. The overall positive autoantibody rate was 84.6% (176/208), the elevated serum IgG (>1 × ULN) rate was 40.9% (85/208), and the elevated serum globulin rate (>1 × ULN) was 31.7% (66/208). The median course of identifiable liver disease at inclusion was 20.0 (8.0, 90.0) days and the median follow-up time was 23.0 (12.0, 36.0) months.

### The etiology and classification of AIH-like liver disease in children

**Figure 1:**
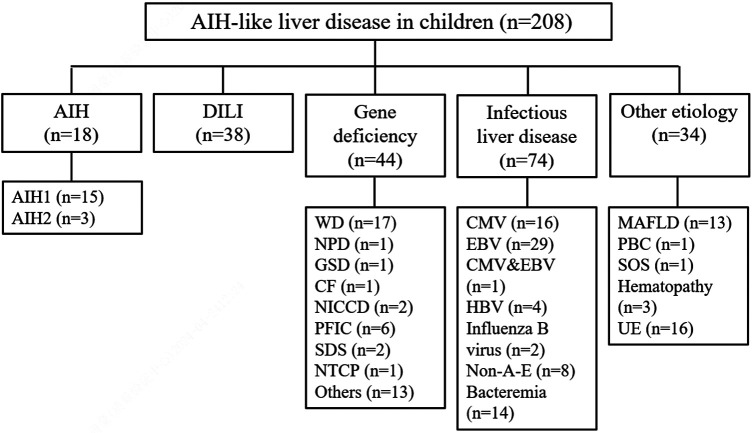
The etiology and classification of AIH-like liver disease in children. AIH, autoimmune hepatitis; DILI, drug-induced liver injury; WD, Wilson's disease; NPD, niemann-pick disease; GSD, glycogen storage disease; CF, cystic fibrosis; NICCD, neonatal intrahepatic cholestasis caused by citrin deficiency; PFIC, progressive familial intrahepatic cholestasis; SDS, shwachman-diamond syndrome; NTCP, sodium taurocholate co-transporting polypeptide deficiency; CMV, cytomegalovirus; EBV, Epstein-Barr virus; HBV, hepatitis B virus; non-A-E, non-A, non-B, non-C, non-D, non-E hepatitis; MAFLD, metabolic dysfunction-associated fatty liver disease; PBC, primary biliary cholangitis; SOS, soidal obstruction syndrome; UE, unknown etiology.

The patients were divided into 5 groups according to the diagnostic criteria for etiological factors. There were 18 patients in the AIH group, 38 patients in the DILI group, 44 patients in the gene deficiency group, 74 patients in the infectious liver disease group, and 34 patients in the other etiology group. The specific causes are shown in Figure 1.

### Comparison of demographic data, manifestations and autoantibodies between the AIH group and the other four groups

As shown in Table 1, there was no significant difference in age or sex among the five groups at the time of initial diagnosis (*p *> 0.05). The recognizable course of liver disease in the AIH group was significantly longer than that in the DILI group at the initial diagnosis (*p *= 0.007), but the follow-up time was significantly longer in the AIH group than in the other four groups after multiple testing correction (*p *< 0.0125).

**Table 1:** Comparison of demographic data, manifestation and autoantibodies between the AIH group and the other four groups.

Characteristics	AIH (*n* = 18)	DILI (*n* = 38)	Gene deficiency (*n* = 44)	Infectious liver disease (*n* = 74)	Other etiology (*n* = 34)	*p*-value
Sex, *n* (%)						
Female	10 (55.6)	20 (52.6)	23 (52.3)	36 (48.6)	15 (44.1)	0.918
Male	8 (44.4)	18 (47.4)	21 (47.7)	38 (51.4)	19 (55.9)	
Age (months)	56.0 (21.5, 102.3)	43.5 (16.3, 108.0)	45.5 (14.3, 99.8)	49.5 (14.5, 84.0)	74.5 (32.3, 122.0)	0.331
Recognizable course (days)	60.0 (10.0, 228.1)	12.0 (4.0, 30.0)**	75.0 (30.0, 262.5)	15.0 (7.0, 28.5)*	75.0 (17.0, 338.8)	<0.001^†^
Follow-uptime (months)	59.0 (27.5, 78.8)	23.5 (18.0, 35.3)**	17.0 (3.0, 29.0)**	21.0 (6.0, 36.0)**	18.0 (6.0, 34.5)**	<0.001^†^
Manifestation, *n* (%)						
Jaundice	8 (44.4)	19 (50.0)	24 (54.5)	11 (14.9)**	7 (20.6)	<0.001^†^
Ventosity	4 (22.2)	3 (7.9)	17 (38.6)	6 (8.1)	6 (17.6)	<0.001^†^
Ascites	2 (11.1)	0 (0.0)	11 (25.0)	1 (1.4)	1 (2.9)	<0.001^†^
Edema	1 (5.6)	1 (2.6)	12 (27.3)	1 (1.4)	0 (0.0)	<0.001^†^
Encephalopathy	1 (5.6)	0 (0.0)	6 (13.6)	2 (2.7)	1 (2.9)	0.034^†^
Fatigue	4 (22.2)	6 (15.8)	8 (18.2)	6 (8.1)	2 (5.9)	0.207
Malaise	3 (16.7)	8 (21.1)	8 (18.2)	7 (9.5)	4 (11.8)	0.463
Pruritus	1 (5.6)	4 (10.5)	4 (9.1)	2 (2.7)	0 (0.0)	0.182
Bellyache	3 (16.7)	3 (7.9)	4 (9.1)	5 (6.8)	4 (11.8)	0.720
Hemorrhage	1 (5.6)	0 (0.0)	1 (2.3)	1 (1.4)	0 (0.0)	0.491
Autoantibody positive, *n* (%)						
ANA = 1:100	0 (0.0)	20 (52.6)**	11 (25.0)*	31 (41.9)**	16 (47.1)**	0.001^†^
ANA ≥ 1:320	16 (88.9)	7 (18.4)**	11 (25.0)**	12 (16.2)**	6 (17.6)**	<0.001^†^
Anti-LKM1	3 (16.7)	0 (0.0)*	0 (0.0)*	0 (0.0)**	0 (0.0)*	<0.001^†^
SMA	2 (11.1)	0 (0.0)	0 (0.0)	0 (0.0)*	0 (0.0)	<0.001^†^
Anti-LC1	3 (16.7)	0 (0.0)*	1 (2.3)	3 (4.1)	0 (0.0)*	0.014^†^
Ro-52	5 (27.8)	4 (10.5)	1 (2.3)**	4 (5.4)**	1 (2.9)**	0.005^†^
AMA-2M	1 (5.6)	1 (2.6)	2 (4.5)	0 (0.0)	0 (0.0)	0.279
AMA-2M-3E	0 (0.0)	0 (0.0)	2 (4.5)	1 (1.4)	0 (0.0)	0.367
Anti-SLA	0 (0.0)	0 (0.0)	1 (2.3)	2 (2.7)	1 (2.9)	0.817
GP210	1 (5.6)	0 (0.0)	0 (0.0)	3 (4.1)	1 (2.9)	0.451
SP100	0 (0.0)	0 (0.0)	0 (0.0)	0 (0.0)	0 (0.0)	–

AIH, autoimmune hepatitis; DILI, drug-induced liver injury; ANA, antinuclear antibodies; anti-LKM1, antibodies to liver-kidney microsomal-1; SMA, smooth muscle antibody; anti-LC1, antibodies to liver cytosol type1; Ro52, Ro52 antibodies; AMA, antimitochondrial antibodies; anti-SLA, antibodies to soluble liver antigen; GP210, nuclear membrane glycoprotein 210 antibodies; SP100, soluble acidic nuclear protein 100 antibodies.

* Compared to the AIH group, *p* < 0.05.

** Compared to the AIH group, *p* < 0.0125 (Bonferroni correction).

^†^ Among the five groups, *p* < 0.05.

Other than the higher proportion of jaundice in the AIH group compared to the infectious liver disease group (*p *= 0.005), there were no significant differences in the main clinical manifestations between the AIH group and the other four groups (*p *> 0.0125).

In addition, the proportion of individuals with ANA ≥ 1:320 was significantly higher in the AIH group compared to the other four groups after multiple testing correction (*p *< 0.0125), while patients with positive anti-LKM1 (*n* = 3) and SMA (*n* = 2) were only observed in the AIH group. The positive anti-LC1 rates in the AIH group were higher than those in other four groups, but this was not statistically significant after multiple testing correction (*p *= 0.029, 0.07, 0.052, and 0.037, respectively); the positive rate for Ro52 was significantly higher than that in the gene deficiency group, infectious liver disease group and the other etiology group (*p *< 0.0125*)*. The positive rates for AMA-2M, AMA-2M-3E, anti-SLA, GP210, and SP100 were similar in each group (*p* > 0.05); the other four groups were more likely to have ANA = 1:100 and other autoantibodies which were weakly positive. All patients in AIH group showed positive autoantibodies, and there were no patient of serologically negative AIH.

### Comparison of laboratory results between the AIH group and the other four groups

As shown in Table 2, during the initial diagnosis, there were no significant differences in blood routine, elevated IgM (>1 × ULN) rate or elevated IgA (>1 × ULN) rate between the AIH group and the other four groups (*p *> 0.0125); the serum IgG and globulin levels were significantly higher in the AIH group than those in the other four groups after multiple testing correction (*p *< 0.0125). There were only three patients with serum IgG levels within the normal range for their respective age groups. Two of these patients had been diagnosed with AIH-1 (ANA positive at 1:1,000 in both patients), and the remaining patient had been diagnosed with AIH-2 (anti-LKM1 positive).

**Table 2:** Comparison of laboratory results between the AIH group and the other four groups.

Characteristic	AIH (*n* = 18)	DILI (*n* = 38)	Gene deficiency (*n* = 44)	Infectious liver disease (*n* = 74)	Other etiology (*n* = 34)	*p*-value
WBC (×10^9^/L)	8.3 (6.6, 10.8)	7.8 (5.6, 10.5)	7.3 (5.2, 10.5)	8.1 (5.9, 11.1)	8.4 (6.3, 9.7)	0.814
Hb (g/L)	133.0 (121.0, 139.3)	125 (120.3, 131)	126 (117, 135.8)	126 (118, 132)	124 (115, 132)	0.281
PLT (×10^9^/L)	272 (214.3, 324.3)	278.5 (209.5, 345.8)	234.5 (142.5, 322.3)	279 (213, 390.3)	306 (209, 342)	0.091
IgG (g/L)	22.8 ± 10.5	11.9 ± 5.7**	13.8 ± 6.2**	13.9 ± 6.4**	11.7 ± 3.1**	<0.001^†^
IgM > 1 ULN	2 (11.1)	0 (0.0)	14 (31.8)	9 (12.2)	3 (8.8)	0.001^†^
IgA > 1 ULN	2 (11.1)	0 (0.0)	13 (29.5)	10 (13.5)	1 (2.9)	<0.001^†^
GLB (g/L)	38.5 (31.5, 46.6)	26.6 (24.1, 30.8)**	29.5 (23.2, 38.2)**	31.8 (24.3, 36.6)**	26.8 (24.8, 33.2)**	0.002^†^
ALT (U/L)	313 (172.9, 686.8)	533 (168.6, 1,427.0)	280.9 (167.5, 335.8)	287.6 (134.8, 570.3)	262.5 (158.8, 484.5)	0.073
AST (U/L)	440.5 (229.3, 806.8)	679 (168.5, 1,087.3)	353.3 (224.8, 427.8)	252 (134.3, 533.8)	261.5 (121, 489.5)	0.011^†^
TB (µmol/L)	48.4 (13.1, 147.6)	16.6 (6.1, 121.3)	51.1 (10.7, 188.6)	9.8 (4.9, 21.2)**	8.8 (5.0, 26.1)**	<0.001^†^
DB (µmol/L)	38.6 (4.9, 121.1)	6.0 (2.1, 106.4)	40.0 (4.7, 143.9)	4.7 (1.8, 17.6)**	3.7 (1.8, 14.7)**	<0.001^†^
GGT (U/L)	104 (40.5, 153.3)	81.5 (37.8, 127.5)	135 (47.3, 194.5)	62 (27.8, 199.9)	70.5 (39.5, 137.5)	0.163
ALP (U/L)	289 (197.5, 373.8)	254 (208, 355.3)	311 (205, 467.8)	232 (170.0, 321.3)	245.5 (203, 344.5)	0.182
LDH (U/L)	339 (251.3, 565.5)	340.5 (287.8, 472.8)	307 (276.3, 414.3)	432 (329.3, 547.5)	316.5 (273.8, 376.5)	0.002^†^
TC (mmol/L)	3.5 (3.0, 4.1)	3.8 (3.1, 4.2)	3.9 (3.2, 4.3)	3.4 (2.9, 4.1)	3.9 (3.4, 4.6)	0.210
ALB (g/L)	32.6 ± 6.9	41.4 ± 5.0**	34.5 ± 8.8	41.1 ± 3.8**	44.0 ± 6.0**	<0.001^†^
PT (s)	16.2 (14.5, 18.9)	13.7 (13.0, 14.5)**	17.0 (13.2, 24.5)	13.7 (12.7, 14.6)**	13.6 (12.6, 14.8)**	<0.001^†^
INR	1.30 (1.1, 1.6)	1.0 (1.0, 1.1)**	1.4 (1.0, 2.3)	1.0 (1.0, 1.2)**	1.0 (0.9, 1.2)**	<0.001^†^

AIH, autoimmune hepatitis; DILI, drug-induced liver injury; WBC, white blood cell; Hb, hemoglobin; PLT, blood platelets; IgG, immunoglobulin G; IgA, immunoglobulin A; IgM, immunoglobulin M; GLB, serum globulin; ULN, upper limit of normal; ALT, alanine aminotransferase; AST, aspartate aminotransferase; TB, total bilirubin; DB, direct bilirubin; GGT, gamma-glutamyl transpeptidase; ALP, alkaline phosphatase; LDH, lactate dehydrogenase; TC, total cholesterol; ALB, albumin; PT, prothrombin time; INR, international normalized rate.

* Compared to the AIH group, *p < *0.05.

** Compared to the AIH group, *p < *0.0125 (Bonferroni correction).

^†^ Among the five groups, *p < *0.05.

TB and DB levels were significantly higher in the AIH group than those in the infectious liver disease group and the other etiology group (*p *< 0.0125); there were no significant differences in serum albumin, PT, or INR levels between the AIH group and gene deficiency group (*p* > 0.0125). However, when compared to the DILI group, the infectious liver disease group, and the other etiology group, there were significant statistical differences observed (albumin was significantly lower, PT was significantly prolonged, and INR was significantly higher, *p *< 0.0125).

### Comparison of histological characteristics between the AIH group and the other four groups

As shown in Table 3, a total of 72 children received liver biopsies. The proportion of portal lymphoplasmacytic infiltration in the AIH group was significantly higher than that in the other 4 groups after multiple testing correction (*p *< 0.0125). The proportion of lobular hepatitis with more than moderate interface hepatitis, and the proportion of lobular hepatitis with lymphoplasmacytic infiltration were significantly higher in the AIH group than those in the other four groups (*p *< 0.0125). The proportions of lobular hepatitis with emperipolesis, hepatocellular rosettes or portal-based fibrosis were higher in the AIH group than those in the other four groups, with no significant difference after multiple testing correction (*p > *0.0125).

**Table 3:** Comparison of histological characteristics between the AIH group and the other four groups.

Characteristics	AIH (*n* = 6)	DILI (*n* = 16)	Gene deficiency (*n* = 18)	Infectious liver disease (*n* = 16)	Other etiology (*n* = 16)	*p*-value
Portal hepatitis, *n* (%)						
Lymphoplasmacytic infiltration	4 (66.7)	1 (6.3)**	1 (5.6)**	1 (6.3)**	1 (6.3)**	<0.001^†^
Biliary tract injury	1 (16.7)	1 (6.3)	4 (22.2)	1 (6.3)	2 (12.5)	0.593
Cholestasis	2 (33.3)	8 (50.0)	10 (55.6)	2 (12.5)	0 (0.0)	0.001^†^
Lobular hepatitis, *n* (%)						
More than moderate interface hepatitis	4 (66.7)	1 (6.3)**	0 (0.0)**	0 (0.0)**	0 (0.0)**	<0.001^†^
Lymphoplasmacytic infiltration	4 (66.7)	1 (6.3)**	1 (5.6)**	1 (6.3)**	1 (6.3)**	<0.001^†^
Rosettes	1 (16.7)	0 (0.0)	2 (11.1)	0 (0.0)	0 (0.0)	0.158
Emperipolesis	2 (33.3)	0 (0.0)*	1 (5.6)	1 (6.3)	0 (0.0)*	0.030^†^
Portal-based fibrosis	4 (66.7)	6 (37.5)	8 (44.4)	6 (37.5)	8 (50.0)	0.731

AIH, autoimmune hepatitis; DILI, drug-induced liver injury.

* Compared to the AIH group, *p < *0.05.

** Compared to the AIH group, *p < *0.0125 (Bonferroni correction).

^†^ Among the five groups, *p < *0.05.

### The AIH scoring system's diagnostic performance

**Figure 2:**
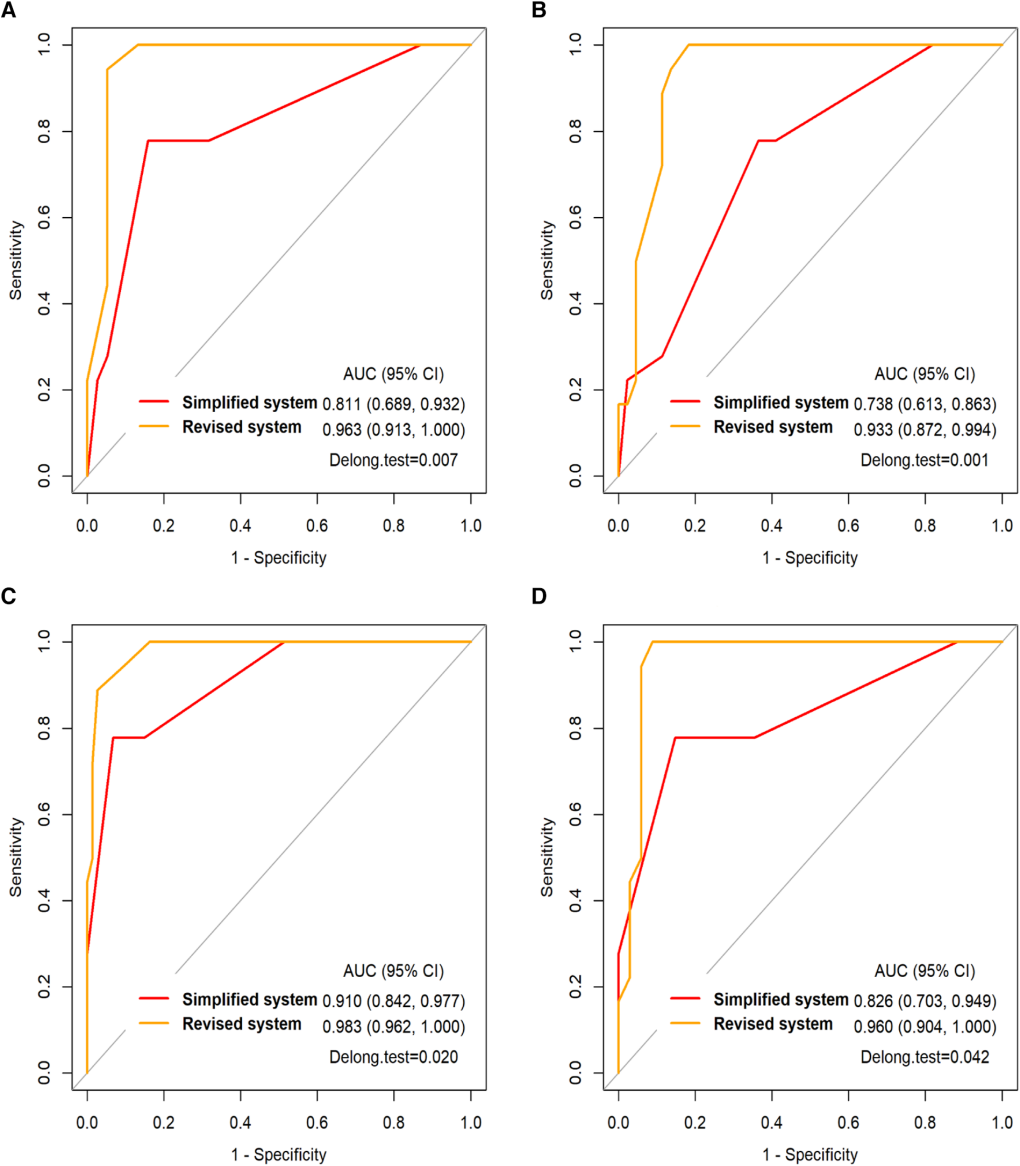
(**A**) the ROC curve of the simplified and revised systems for the AIH in DILI group and (**B**) in gene deficiency group and (**C**) in infectious liver disease group and (**D**) in other etiology group. AUC, the area under the ROC curve.

As shown in Table 4, the simplified and revised system in the AIH group were significantly higher than those in the other 4 groups (*p* < 0.0125). As shown in Figure 2, the area under the ROC curve (AUC) for each group reveals that both the simplified (AUC > 0.73) and the revised systems (AUC > 0.93) for AIH have good diagnostic performance. The difference in the area under the ROC curve suggests that the revised systems outperforms the simplified systems in terms of diagnostic accuracy, as confirmed by the Delong test (*p* < 0.05).

**Table 4:** Comparison of AIH score systems, treatment and outcome between the AIH group and the other four groups.

Characteristics	AIH (*n* = 18)	DILI (*n* = 38)	Gene deficiency (*n* = 44)	Infectious liver disease (*n* = 74)	Other etiology (*n* = 34)	*p*-value
Score systems						
Simplified system	6.0 (5.5, 7.3)	4.0 (4.0, 5.0)**	4.0 (4.0, 6.0)**	4.0 (2.0, 4.0)**	4.0 (4.0, 5.0)**	<0.001^†^
Revised system	17.5 (16.0, 19.3)	8.0 (6.0, 10.0)**	9.0 (7.3, 12.8)**	10.0 (5.8, 13.0)**	9.5 (7.8, 12.0)**	<0.001^†^
Treatment, *n* (%)						
Corticosteroids	18 (100)	16 (42.1)**	13 (29.5)**	16 (21.6)**	4 (11.8)*	<0.001^†^
Immunomodulator	14 (77.8)	0 (0.0)**	0 (0.0)**	2 (2.7)**	0 (0.0)**	<0.001^†^
Outcome, *n* (%)						
Recovery	0 (0.0)	31 (81.6)**	1 (2.3)	55 (74.3)**	11 (32.4)**	<0.001^†^
Chronicity	18 (100)	6 (15.8)**	43 (97.7)	15 (20.3)**	22 (64.7)	<0.001^†^
Cirrhosis	7 (38.9)	0 (0.0)**	28 (63.6)	2 (2.7)**	4 (11.8)	<0.001^†^
Death	1 (5.6)	1 (2.6)	13 (29.5)*	1 (1.4)	1 (2.9)	0.008^†^

AIH, autoimmune hepatitis; DILI, drug-induced liver injury.

* Compared to the AIH group, *p < *0.05.

** Compared to the AIH group, *p < *0.0125 (Bonferroni correction).

^†^ Among the five groups, *p < *0.05.

### Comparison of treatment and outcome between the AIH group and the other four groups

All children diagnosed with AIH at our center received immunosuppressive therapy, and the proportion of those who had accepted immunosuppressive therapy in the AIH group was significantly higher than those in the other 4 groups after multiple testing correction (*p *< 0.0125). Four children with AIH received corticosteroid therapy alone. Among them, two patients were lost to follow-up (confirmed in 2013), one child was confirmed in the adult (i.e., non-pediatric) department and one child died. After enduring two surgeries for intestinal obstruction, this child developed severe malnutrition and a low blood cell count, which prevented him from tolerating immunosuppressive therapy. Nevertheless, he managed to maintain biochemical stability through corticosteroid therapy alone. The other 14 patients were treated with prednisone (1–2 mg/kg/day) combined with azathioprine (1–2 mg/kg/day). Once biochemical remission was achieved after the initial treatment (usually within 4–6 weeks), the dose of prednisone was gradually reduced, with a general maintenance dose of 5–10 mg/day. Azathioprine was maintained at the aforementioned dose, typically 1 mg/kg. Two patients relapsed and developed refractory AIH, for which they were switched to methylprednisolone (15–20 mg/day) combined with mycophenolate mofetil (CellCept: 33 mg/kg). The biochemical remission rate for children with AIH at our center is 94.4%.

As shown in Table 4, the liver disease recovery rate in the AIH group was similar to that in the gene deficiency group (*p *= 1.000), but it was lower than that in the DILI group, infectious liver disease group, and the other etiology group (*p *< 0.0125). The chronicity and cirrhosis rates for liver disease in the AIH group were similar to those in the gene deficiency group and the other etiology group (*p *> 0.0125), but were higher than those in the DILI group and the infectious liver disease group (*p *< 0.0125); the mortality rate in the AIH group was lower than that in gene deficiency group, but this was not statistically significant after multiple testing correction (*p *= 0.04).

A total of 17 deaths occurred. The gene deficiency group accounted for 13 deaths, of which six patients succumbed to liver failure resulting from decompensation of cirrhosis, while another six died from infectious shock triggered by pneumonia following cirrhosis. Additionally, one patient passed away due to liver transplant failure. The AIH group accounted for one death, and the reason was described above. The DILI group accounted for one death, of infectious shock complicated with aplastic anemia. Another death was in the infectious liver disease group, and due to severe hepatitis complicated by liver failure. The other etiology group had one death, of complications after liver transplant.

### Recommendations for AIH diagnostic procedures

**Figure 3:**
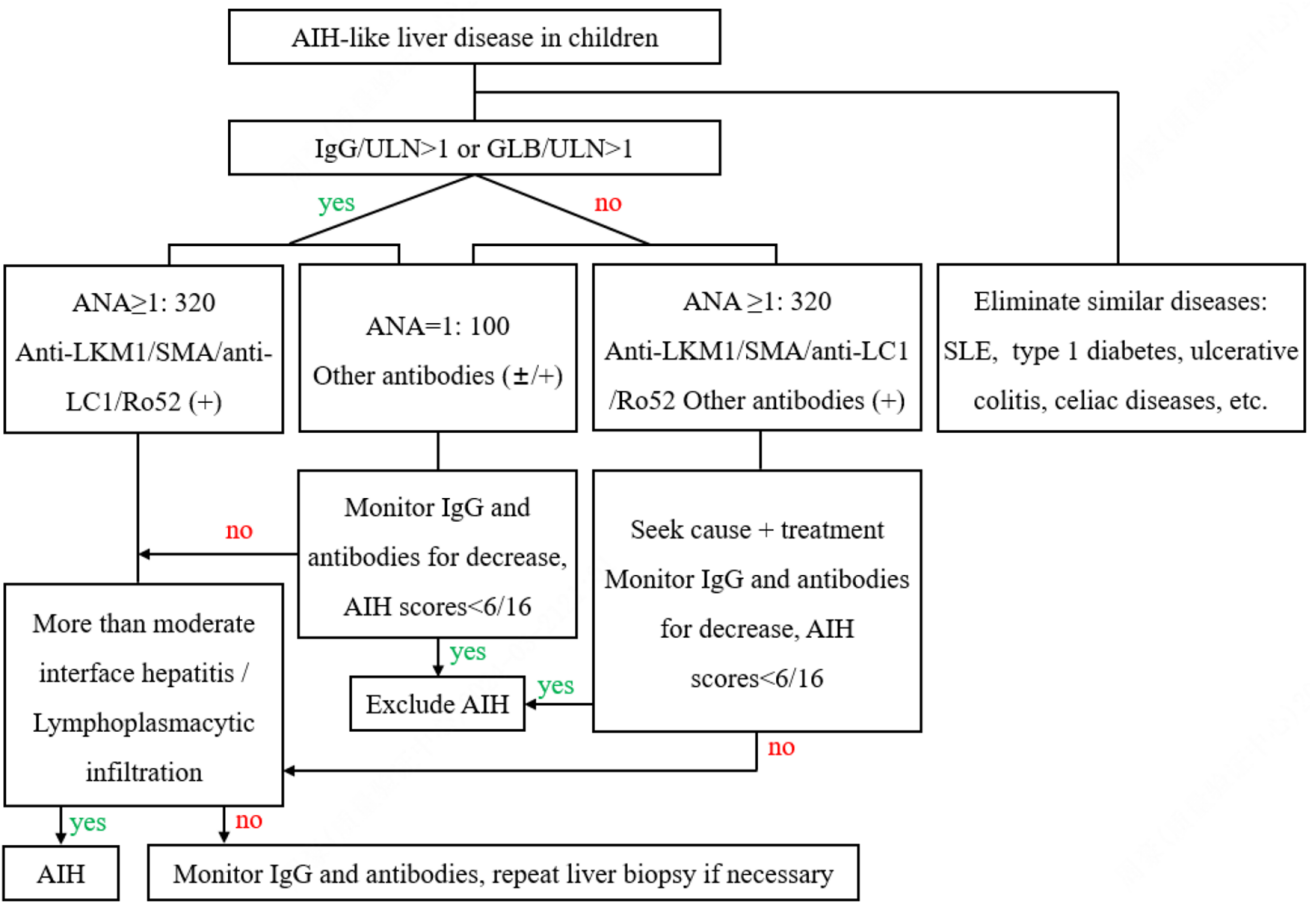
AIH diagnostic procedures and clinical pathways. AIH, autoimmune hepatitis; SLE, systemic lupus erythematosus; IgG, immunoglobulin G; GLB, seroglobulin; ULN, upper limit of normal; ANA, antinuclear antibodies; anti-LKM1, antibodies to liver-kidney microsomal1; SMA, smooth muscle antibodies; anti-LC1, antibodies to liver cytosol type1; Ro-52, Ro-52 antibodies.

Based on the above results, we proposed AIH diagnostic procedures and clinical pathways, as shown in Figure 3.

## Discussion

AIH proportion varies significantly depending on the region, age of onset and sex. The estimated prevalence rate is 1.28–15.65/100,000, and the prevalence rate among children is significantly lower than that in adults (16). Over the past 10 years, only 18 patients with AIH in children have been diagnosed at our center. We also found that other diseases may have clinical manifestations similar to those of AIH, such as DILI (38/208), gene deficiency disease (44/208) and infectious liver disease (74/208). This article retrospectively compares the differences between AIH and other AIH-like liver diseases at baseline, in hopes of providing a reference for early diagnosis of AIH in clinical practice.

The clinical manifestations of AIH in children are not specific, and may include symptoms such as fatigue, jaundice and itching. About 40% of patients with AIH show the same symptoms as acute viral hepatitis; another 40% of patients among children show signs of chronic liver disease, such as progressive fatigue, intermittent jaundice and weight loss. These patients may even have decompensated manifestations of cirrhosis such as hepatosplenomegaly, ascites and/or gastrointestinal bleeding (17, 18). Similar to other literature, our results show no significant differences in the manifestations between the AIH group and the other four groups. Only the proportion of jaundice in the AIH group is higher than that of the infectious liver disease group. The main cause is believed to be that most children with infectious diseases in this group are infected with EBV (40.5%, 30/74). Acute EBV infection typically manifests as non-jaundice hepatitis, and jaundice is rare (19).

Although positive autoantibodies are also commonly found in other liver diseases such as viral hepatitis and DILI, positive autoantibodies still strongly support diagnosis of AIH (20, 21). ANA or SMA positive and anti-LKM1 positive are characteristic autoantibodies for type 1 and type 2 AIH, respectively (22). We found that the highest ANA ≥ 1:320 rate was in the AIH group, while ANA = 1:100 and other weak positive autoantibodies were more likely to be present in the non-AIH group. Low-titer autoantibodies are found in patients with hepatolenticular degeneration, who are not directly associated with liver function impairment, steatosis and fibrosis (23). Antibodies that are more specific to autoimmune hepatitis, such as anti-LKM1, are not present in patients with MAFLD (5). This is consistent with the results of this article, with only the AIH group showing positive anti-LKM1. Other autoantibodies also contribute to the diagnosis of AIH (24). We found that the positive rates of anti-LC1 (indicating type 2 AIH) and Ro-52 in the AIH group were higher than those in the other four groups. However, they were also found in other patients, making them less specific. The results indicated that the presence of ANA ≥ 1:320, either alone or in combination with SMA or anti-LKM1 positivity, strongly supported the diagnosis of AIH. On the other hand, patients who exhibited positivity for only anti-LC1 or Ro-52 were recommended for follow-up monitoring to avoid missed diagnoses. Although anti-SLA are found in approximately 10%–20% of patients with AIH, they have a high specificity for the diagnosis of AIH (25). Interestingly, all four patients with positive anti-SLA at our center were excluded from AIH. They were under three months old, and these antibodies are believed to be maternally transmitted (26). Approximately 10% of patients with AIH are seronegative AIH (27), yet our center has not encountered any patients of serologically negative AIH. This may be attributed to the fact that the diagnosis of most cases in our study is primarily based on the AIH scoring systems.

The increase in serum IgG and gamma globulin levels is an important sign of serum antibody-positive AIH (2, 24). In our study, we included the serum globulin level as a criterion for diagnosing AIH-like liver disease, as it is a routinely performed clinical test that offers early clinical insights into potential elevations in gamma globulin (28). The results showed that the levels of serum IgG and globulin in the AIH group were significantly higher than those in the other four groups. Although some diseases, such as chronic viral hepatitis or cirrhosis caused by other etiologies, can also lead to increased serum globulin and IgG levels (3), some scholars have reported that the serum IgG levels in patients with AIH are significantly higher than those in non-autoimmune hepatitis and cirrhosis patients. This finding suggested that a high serum IgG level is the best indicator to distinguish autoimmune hepatitis from other AIH-like liver diseases (29). The levels of serum IgA and IgM in patients with AIH are generally normal (30). Our results showed that there was no significant difference between the AIH group and the other four groups in terms of increases in IgM and IgA rates, which is consistent with previous literature.

IgG4-related disease (IgG4-RD) is an immune-mediated chronic inflammatory disease which is accompanied by fibrosis that can affect most of the body (31). When IgG4-RD only involves the liver and/or biliary system, it needs to be differentiated from AIH. Imaging tools can help identify typical lesions of IgG4-RD, such as diffuse or focal enlargement of organs and retroperitoneal fibrosis (32). Pathologically, although IgG4-RD exhibits histological similarities to AIH, significant infiltration of IgG4-positive plasma cells is helpful for diagnosis (33, 34). Only five patients with AIH in our center have had their IgG4 levels tested, and none showed abnormal results.

Overlap syndrome AIH with primary sclerosing cholangitis (PSC) is reportedly more prevalent in childhood, affecting approximately 33% of children with autoimmune liver disease, whereas only 1.7 to 10% of adults are affected (35). In our study, two children with cholestasis and suspected of having overlap syndrome AIH with PSC had elevated GGT and ALP levels. Nevertheless, despite undergoing Magnetic Resonance Cholangiopancreatography, no specific imaging features indicative of PSC, such as bile duct stenosis or dilatation, were observed.

The levels of albumin, INR or PT reflect the synthetic function of the liver, and these are also the prognostic indicators for liver disease (36). The degree of impairment of the synthetic function of the liver (prolonged PT, elevated INR, and hypoalbuminemia) in the AIH group at our center is comparable to that in the gene deficiency group, but much more severe than that in the DILI group, infectious liver disease group, and other etiology group. Some studies have reported that patients with AIH typically show elevated aminotransferase levels, with approximately half of the patients having impaired synthetic function, namely low albumin levels and elevated international normalized rates (37), which is consistent with the findings of this study.

The main histological features of AIH are more than moderate interface hepatitis and lymphoplasmacytic infiltration (3, 38), which are not highly specific. We found that portal lymphoplasmacytic infiltration, lobular inflammation combined with more than moderate interface hepatitis and lymphoplasmacytic infiltration are the main features of AIH. Other chronic liver diseases such as DILI, viral hepatitis, and Wilson's disease may also be comorbid with interface hepatitis (39), which is usually mild or moderate. The lobular hepatitis with lymphoplasmacytic infiltration is considered the characteristic histological feature of acute-onset AIH, which is not included in the revised and simplified IAIHG systems (2, 40). Lymphoplasmacytic infiltration is another characteristic feature of AIH (18), but only two patients showed lymphoplasmacytic infiltration in AIH group. Of course, the number of liver biopsies performed in this research center is relatively small. In the future, more patients are needed to draw more definitive conclusions.

The AIH scoring systems have had good sensitivity and specificity in most patients (41). In our study, the simplified and revised systems had good diagnostic efficiency for AIH. Some studies have shown that compared with the simplified system, the revised system has higher sensitivity (100% vs. 95%), lower specificity (73% vs. 90%), and lower accuracy (82% vs. 92%) for diagnosis (42). However, our results showed that the revised system's diagnostic accuracy was superior to that of the simplified system. Some scholars have suggested that the simplified system is more applicable to patients with typical performance, while the revised system is more applicable to patients with atypical performance, which may be the reason why the revised system's diagnostic accuracy surpassed that of the simplified system in our study (40). Only 34.6% (72/208) of the children in this study received liver biopsies at their initial visit, so most of the children did not receive scores for the liver biopsy item, which may have biased the scoring results.

During the follow-up period, none of the patients in the DILI group progressed to cirrhosis, while 38.9% of the children with AIH eventually developed cirrhosis, which is consistent with previous literature (43, 44). Many gene deficiencies reported in the literature have a tendency to progress to cirrhosis, including WD (45), This finding is consistent with our research, which demonstrated that the cirrhosis rate in the gene deficiency group similar to that in the AIH group. According to the literature, the 10-year survival rate for AIH is 79%–98%, the 20-year survival rate is 77%–90%, and the 30-year survival rate is 55% (46). The mortality rate (1/18, due to non-AIH diseases) in the AIH group was not high, which indicated that the prognosis of AIH is generally favorable, highlighting the importance of early diagnosis and effective management in maintaining a positive outcome for patients.

At present, the diagnosis, examination, and follow-up management system for adult AIH has been well established, but research on pediatric patients remains limited (47). Based on the results of this study, we proposed a diagnostic process for AIH, which may help general pediatricians to identify and diagnose AIH.

## Advantages and limitations of this study

The advantage of this study lies in its extended follow-up period, with some patients being monitored for up to 10 years. However, several limitations must be acknowledged. Firstly, this study is retrospective and the number of patients with AIH is small. Secondly, the absence of radiological data poses another constraint in the study. Thirdly, the small number of patients receiving liver biopsy may have introduced some bias into the analysis of the liver histopathological characteristics and the evaluation of the AIH scoring system's efficacy.

## Conclusion

The diagnosis of AIH is rather challenging. This article retrospectively analyzes the differences between AIH and other causes of AIH-like liver disease at baseline. We have proposed that ANA ≥ 1:320, being positive for anti-LKM1 or SMA (with anti-LC1 or Ro-52 positivity serving as auxiliary diagnostic significance), elevated serum IgG or globulin levels are helpful for early identification of AIH. Moreover, the presence of lobular hepatitis with more than moderate interface hepatitis and lymphoplasmacytic infiltration contribute to the diagnosis of AIH. The proposed diagnostic process for AIH would help general pediatricians with early recognition of AIH.

## Data Availability

The original contributions presented in the study are included in the article/Supplementary Material, further inquiries can be directed to the corresponding authors.
